# The pharmacological effect of BGC20-1531, a novel prostanoid EP_4_ receptor antagonist, in the Prostaglandin E_2_ human model of headache

**DOI:** 10.1007/s10194-011-0358-9

**Published:** 2011-06-17

**Authors:** Maria Antonova, Troels Wienecke, Karen Maubach, Emma Thomas, Jes Olesen, Messoud Ashina

**Affiliations:** 1 Danish Headache Center and Department of Neurology, Faculty of Health Sciences, Glostrup Hospital, University of Copenhagen, Ndr. Ringvej 67, building 16, 2600 Glostrup, Denmark; 2 BTG, 5 Fleet Place, London, EC4M 7RD UK; 3 Danish Headache Center and Department of Neurology, Faculty of Health Sciences, Glostrup Hospital, University of Copenhagen, Ndr. Ringvej 67, building 14, 2600 Glostrup, Denmark

**Keywords:** Prostaglandin E_2_, EP_4_ receptor antagonist, BGC20-1531, Vasodilatation, Headache

## Abstract

Using a human Prostaglandin E_2_ (PGE_2_) model of headache, we examined whether a novel potent and selective EP_4_ receptor antagonist, BGC20-1531, may prevent headache and dilatation of the middle cerebral (MCA) and superficial temporal artery (STA). In a three-way cross-over trial, eight healthy volunteers were randomly allocated to receive 200 and 400 mg BGC20-1531 and placebo, followed by a 25-min infusion of PGE_2_. We recorded headache intensity on a verbal rating scale, MCA blood flow velocity and STA diameter. There was no difference in headache response or prevention of the dilation of the MCA or the STA (*P* > 0.05) with either dose of BGC20-1531 relative to placebo, although putative therapeutic exposures were not reached in all volunteers. In conclusion, these data suggest that the other EP receptors may be involved in PGE_2_ induced headache and dilatation in normal subjects.

## Introduction

The arachidonic acid metabolite prostaglandin E_2_ (PGE_2_) plays an important physiological role in the human body including the regulation of vascular tone [1] and modulation of pain [2]. PGE_2_ acts via four different G-protein-coupled receptor subtypes: EP_1_, EP_2_, EP_3_ and EP_4_ [3]. Once activated by PGE_2_, EP_1_ and EP_3_ receptors mediate Ca^2+^ mobilisation and decrease levels of cAMP, which leads to smooth muscle contraction [4]. In contrast, PGE_2_ action on EP_2_ and EP_4_ stimulates adenylate cyclase and thereby causes relaxation of vascular smooth muscles [5]. It has been demonstrated that PGE_2_-mediated vasodilatation of the human middle cerebral (MCA) and meningeal arteries (MMA) occurs primarily due to activation of the EP_4_ receptors and the EP_4_ receptor antagonist, AH 23848, is able to attenuate the PGE_2_ vasodilating response [6]. PGE_2_, as a principal pro-inflammatory prostanoid, plays a role in nociceptive processing [7]. It has both direct activating and sensitizing effects on sensory neurones [8]. Furthermore, increased levels of PGE_2_ caused up-regulation of the EP_4_ receptor subtype in rat sensory dorsal root ganglion (DRG) neurons, but not EP_1_ and EP_3_ receptor subtypes [9]. Given that sensitization of the sensory neurons mediated mainly through the EP_4_ receptors [10] it has been suggested that the prostanoid EP_4_ receptor may be a potential target for the treatment of pain [11].

A novel selective and potent EP_4_ receptor antagonist, BGC20-1531, has been tested in an in vitro human study [12]. BGC20-1531 antagonized PGE_2_-mediated dilatation of human middle cerebral and middle meningeal artery rings, pre-contracted with phenylephrine. It has therefore been suggested that BGC20-1531 has the potential to alleviate the symptoms of migraine pain caused by dilatation of cerebral arteries [12]. A human PGE_2_ model of headache has been developed and has demonstrated that PGE_2_ induces dilatation of cranial arteries and causes headache in healthy subjects [13]. Whether BGC20-1531 can block the PGE_2_ induced responses in humans has previously not been studied.

The aim of the present study was to evaluate the effect of two different single oral doses of the EP_4_ receptor antagonist BGC20-1531 on PGE_2_-induced dilatation of cranial vessels and headache in a randomised, double blind, placebo-controlled, three-way intra-individual crossover study.

## Methods

### Design and subjects

The study was designed as a randomised, double blind, placebo-controlled, three-way intra-individual crossover study. Eight healthy volunteers (5 male and 3 female), mean age 24 years (range 21–30 years) and mean weight 75.7 kg (range 67–93.5 kg) completed the study. Subjects had no history or family history of migraine, or any other type of headache (except episodic tension-type headache less that once a month) and no previous serious somatic, psychiatric or infectious diseases. Physical and neurologic examination, electrocardiography (ECG), clinical-chemical and haematological screenings were done on the day of enrolment.

All subjects were randomly assigned to receive BGC20-1531 200 mg, BGC20-1531 400 mg or placebo, followed 75 min later by an infusion of PGE_2_ at 0.40 μg/kg/min over 25 min on three different days at intervals of at least 1 week. The study drug BGC20-1531 and equivalent placebo were provided by BTG International Ltd, London, UK, and randomised and blinded by the central pharmacy, Herlev Hospital, Denmark. The randomization code was kept in the hospital during the study and the unblinding procedure was first performed after the study was completed. PGE_2_ was purchased from Cayman Pharma, Neratovice, Czech Republic. The dose of PGE_2_ (0.40 μg/kg/min) was selected based on a PGE_2_-induced headache study in healthy volunteers [13].

The study protocol was approved by the Ethics Committee of the Country of Copenhagen (VEK H-D-2008-134), Danish Medical Agency (EudraCT 2008-008713-20) and Danish Data Protection Agency and performed in accordance with the Helsinki Declaration of 1964, as revised in Edinburgh in 2000. The study was registered on http://www.clinicaltrials.gov. All subjects gave written informed consent to participate in the study. The trial was conducted according to the protocol and Good Clinical Practice (GCP), and monitored externally by the GCP unit from the Copenhagen University Hospital.

### Headache intensity and adverse events

To record headache intensity, a 10-point verbal rating scale (VRS) was used, where 0 indicated no headache; 1 indicated a different sensation, pounding or throbbing, but not necessarily painful; 5 indicated moderate headache and 10 indicated worst imaginable headache [14]. Subjects were encouraged to self-report any changes in their well-being during the study. Subjects were questioned about the presence of adverse events (AEs), headache and accompanying symptoms according to the International Headache Classification (IHC) [15] at *T*_−75_, *T*_−30_, *T*_0_ and then every 10th min until *T*_90._ During the out-of-hospital period, defined as a period after discharge and until bedtime, all subjects were carefully instructed to make hourly recordings of headache and accompanying symptoms according IHC [15] and any other AEs. All AEs were classified as related or not related to the study drug by the investigator. Subjects were allowed to take rescue medication of their own choice after consulting the study physician.

### Transcranial Doppler and C-scan

Blood flow velocity was recorded in the middle cerebral artery (V_MCA_) by a Transcranial Doppler (TCD) ultrasonography (2 MHz) with handheld probes (Multidop X; DWL, Sipplingen, Germany) [13]. The recordings were performed bilaterally and simultaneously with measurements of end-tidal partial pressure of pCO_2_ (P_et_CO_2_), obtained with an open mask without any respiratory resistance (ProPac Encore^®^; Welch Allyn Protocol, Beaverton, OR, USA) as previously described [16]. A fixed point was used with the best possible signal along the MCA, as close as possible to the bifurcation of the anterior cerebral artery and MCA. The fix point was marked and noted and was reused in each participant for all recordings. All measurements were done by the same skilled laboratory technician.

A high resolution ultrasound scanner, C-scan (20 MHz, bandwidth 15 MHz; Dermascan C; Cortex Technology, Hadsund, Denmark) was used to measure the diameter of the frontal branch of the left superficial temporal artery (STA) and the left radial artery (RA). All C-scans were performed in the same place as ensured by markings drawn on the skin. The coordinates of the marks were kept for reuse in the following trial days. All measurements within the same study subject were done by the skilled laboratory technician.

### Pharmacokinetics

Blood samples for the plasma concentration of BGC20-1531 were collected at *T*_−75_, *T*_0_, *T*_30_, *T*_60_ and *T*_90_ on each study day in Vacuette^®^ Lithium Heparin 4 ml tubes (Greiner Bio-one, Austria). Samples were immediately stored on ice and then separated by centrifugation at 1,500×*g* and 4°C for 10 min. Two identical aliquots of plasma were transferred in to polypropylene tubes (Sarstedt, Germany) and stored at −25°C until analyzed at Simbec Research Ltd, UK.

### BGC20-1531 analytical methods

Plasma concentration of BGC20-1531 was determined by liquid chromatography with tandem mass spectrometry detection (LC-MS-MS). The analyses was performed using atmospheric pressure ionization with turbo ion spray followed by multiple reaction monitoring (MRM) of the characteristic ion transitions for BGC20-1531 and internal standard.

### Trial procedure

Subjects were required to limit alcohol intake to 2 units per day for 7 days before the first dose and until the trial period was finished and to avoid alcoholic beverages entirely for 2 days prior to and 2 days after each treatment session. Subjects had to abstain from caffeine intake 2 days before the first dosing and until the end of the study and cocoa and chocolate were not allowed 24 h before the dosing day. All subjects were non-smokers. Use of pharmacologic agents apart from oral contraceptives were not permitted. Subjects fasted overnight and reported to the laboratory at 8 a.m. and were confirmed to be headache-free. Subjects rested in the supine position throughout the study period from time −75 min (start of study period, 75 min prior to the infusion) to *T*_90_ (end of study period, 90 min post infusion). The procedures were performed in a quiet room at room temperature between 21 and 24.7°C. Two intravenous catheters Venflon^®^ (Becton Dickton, Sweden) were inserted into the antecubital veins for the PGE_2_ infusion and blood sample collection for BGC20-1531 plasma concentration analysis. The subjects rested for at least 30 min before *T*_−75_ values of flow velocity in the middle cerebral artery (V_MCA_), diameters of STA and RA, mean arterial blood pressure (MAP), heart rate (HR), P_et_CO_2_, transcutaneous arterial oxygen saturation (SAT), ECG, headache score and AEs were recorded.

Following the baseline measurements, subjects were randomized to BGC20-1531 200 mg, BGC20-1531 400 mg or placebo. At *T*_0_, the infusion of PGE_2_ (0.40 μg/kg/min) was initiated by a time and volume controlled infusion pump (Braun Perfuser, Melsungen, Germany). The timing of the infusion ensured that a steady state of BGC20-1531 was reached, as the *T*_max_ was predicted to be approximately 60 min.

All measurements were recorded at *T*_−75_, *T*_−30_, *T*_0_ and then every 10th min until *T*_90_. MAP and HR were measured by an auto-inflatable cuff (ProPac Encore^®^; Welch Allyn Protocol, Beaverton, OR, USA). ECG was obtained continually using Cardiofax V (Nihon-Cohden, Japan) and recorded on paper at time as described above.

### Statistics

Vascular variables are presented as mean ± SD and as mean percentage from baseline. Headache scores are presented as median and quartiles. As we did not record any vascular or headache responses after BGC20-1531 administration during *T*_−75_ to *T*_0_ baseline was defined as *T*_0_ before start of PGE_2_ infusion. Immediate headache was defined as any headache during the in-hospital period (0–90 min) and delayed headache (1.5–11 h) was defined as any headache during the out-of-hospital period. The data were baseline-corrected and the area under the curve for the time period *T*_0_–*T*_90_ (AUC) for V_MCA_, headache score, MAP, HR and PetCO_2_ was calculated, using the trapezium rule [17].

The sample size was calculated based on proven difference between treatments, measured as reduced pain intensity on the VRS at 5% significance (one-sided) with 90% power. We assumed 20% deviation on the VRS for each study subject and 70% reduction of pain intensity was considered to be clinically significant, therefore 8 subjects were included [18].

The primary end-points were differences in the AUC for headache score (AUC_headache score_) between active and placebo arm, placebo versus BGC20-1531 200 mg and placebo versus BGC20-1531 400 mg. The secondary end-points were differences in the AUC for VMCA (AUC_VMCA_), STA (AUC_STA_), RA (AUC_RA_), PetCO_2_ (AUC_PetCO2_), MAP (AUC_MAP_) and HR (AUC_HR_) between placebo and two active treatment arms. To test the statistical difference between the variables we applied a paired, two-way *t* test for vascular data, the Wilcoxon signed ranks test for headache score, and the McNemar test for AEs. To explore possible changes over time for vascular variables we conducted post hoc analysis by repeated measures one-way ANOVA (including the Dunnett post hoc test).

Five percent (*P* < 0.05) was accepted as the level of significance. All analyses were performed with PASW Statistics 18 for Windows (SPSS Inc., Chicago, IL, USA). Post hoc exploratory analyses were performed using GraphPad Prism^®^ (GraphPad Software Inc., CA, USA).

## Results

Eight healthy volunteers completed the study. 11 subjects were enrolled with 3 participants being withdrawn after the first day of dosing. One was withdrawn due to severe chills and shivering during PGE_2_ infusion, another due to an unspecific T-wave inversion in the pre-cordial leads on ECG and the third due to a drop in diastolic blood pressure below 40 mmHg, which was a safety limit according to the study protocol.

### Baseline values

There were no differences in baseline recordings for any variables between placebo and active days. There were no differences in baseline velocity in the middle cerebral artery (V_MCA_) between the left and the right side on all three study days (data not shown).

### Effect of BGC20-1531 on PGE_2_-induced headache

**Fig. 1:**
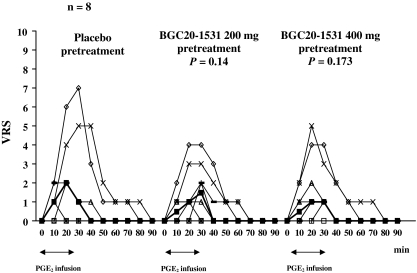
Median (*filled square*) and individual headache scores on a verbal rating scale (VRS) on placebo compared to either pretreatment day with BGC20-1531. The median peak immediate headache score was 2 at *T*_20_ on placebo day, 1.5 at *T*_30_ on BGC20-1531 200 mg day, and 1 at *T*_20_ on BGC20-1531 400 mg day. No difference between AUC headache score on placebo and either BGC20-1531 pretreatment was found (Wilcoxon signed ranks test)

The incidence of immediate and delayed headache is shown in Table 1. There was a large variation in the severity of headache between the subjects on placebo day and we found no difference in area under the curve (AUC) for headache between both pretreatment days and placebo day (BGC20-1531 200 mg: *P* = 0.14; BGC20-1531 400 mg: *P* = 0.173) (Fig. 1).

**Table 1:** Incidence of Prostaglandin E_2_ (PGE_2_)-induced immediate and delayed headache in eight healthy subjects

	Placebo plus PGE_2_	BGC20-1531 200 mg plus PGE_2_	BGC20-1531 400 mg plus PGE_2_
Incidence of immediate headache	6	6	7
Incidence of delayed headache	1	1	1

McNemar test showed no difference in incidence of immediate and delayed headache between placebo and BGC20-1531 200 and 400 mg (*P* > 0.05)

### Effect of BGC20-1531 on velocity of middle cerebral artery

**Fig. 2:**
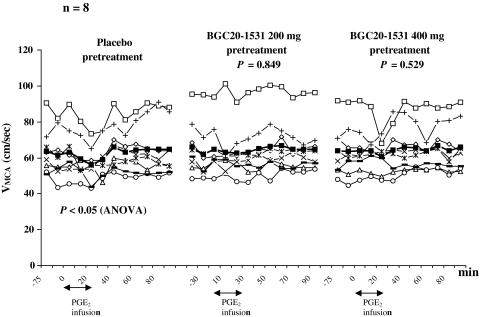
Individual and mean (*filled square*) flow velocities (cm/s) in the middle cerebral arteries (V_MCA_) on placebo day compared with BGC20-1531 before and after Prostaglandin E_2_ (PGE_2_) infusion. There was no difference between AUC_VMCA_ on placebo and BGC20-1531 200 or 400 mg pretreatment (paired *t* test)

We found no difference in the AUC_VMCA_ between placebo and BGC20-1531 200 mg (*P* = 0.849) and 400 mg (*P* = 0.529) (Fig. 2). There was no difference in the AUC for end-tidal partial pressure of pCO_2_ (PetCO_2_) between both pretreatment days and placebo day (BGC20-1531 200 mg: *P* = 0.700; BGC20-1531 400 mg: *P* = 0.712). Explorative ANOVA analysis revealed significant changes over time in V_MCA_ after placebo (*P* < 0.05) but not after BGC20-1531 200 and 400 mg (*P* > 0.05). As expected, post hoc Dunnetts test showed a significant drop in V_MCA_ at *T*_20_ after PGE_2_ infusion on placebo day compared to baseline (*P* < 0.05).

### Effect of BGC20-1531 on diameter of superficial temporal and radial arteries

**Fig. 3:**
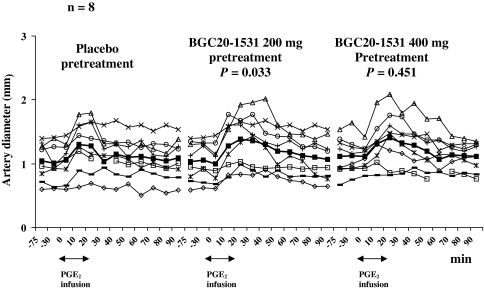
Individual and mean (*filled square*) diameter (mm) in the superficial temporal artery (STA) on placebo day compared with BGC20-1531 before and after Prostaglandin E_2_ (PGE_2_) infusion. There was a difference between AUC_STA_ on BGC20-1531 200 mg pretreatment and placebo, and no difference between AUC_STA_ on placebo and 400 mg pretreatment (paired *t* test)

The superficial temporal artery AUC (AUC_STA_) on BGC20-1531 200 mg day was significantly larger than on placebo day (*P* = 0.033). We found no difference in the AUC_STA_ between placebo and BGC20-1531 400 mg day (*P* = 0.451) (Fig. 3). There was no difference in the radial artery AUC (AUC_RA_) between each pretreatment day compared with placebo day (*P* = 0.678 and *P* = 0.575 on BGC20-1531 200 mg and BGC20-1531 400 mg pretreatment day respectively).

### Peripheral hemodynamics

We found no difference in the AUC for mean arterial blood pressure (AUC_MAP_) between placebo and BGC20-1531 200 mg day (*P* = 0.267) and placebo and BGC20-1531 400 mg day (*P* = 0.450). There was also no difference in the AUC_HR_ on placebo day compared with the AUC_HR_ on BGC20-1531 200 mg (*P* = 0.799) day and 400 mg day (*P* = 0.074).

### Pharmacokinetic profile of BGC20-1531

**Fig. 4:**
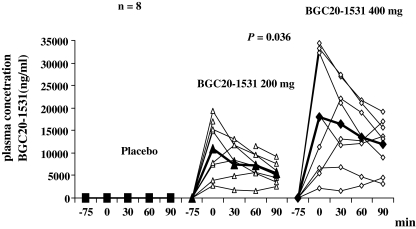
Individual (*open geometric shapes*) and mean (*filled geometric shapes*) plasma concentration of BGC20-1531on placebo and either active day. Significant difference between AUC_PC_ on BGC20-1531 400 mg pretreatment and BGC20-1531 200 mg pretreatment (*P* = 0.036) (paired *t* test)

The highest plasma concentration of BGC20-1531 in our study was detected 75 min after oral administration of BGC20-1531 200 and 400 mg at *T*_0_ (Fig. 4). No plasma BGC20-1531 was detected in samples taken on placebo day. The AUC plasma concentration on pretreatment with BGC20-1531 400 mg was significantly larger compared to the AUC plasma concentration on BGC20-1531 200 mg (*P* = 0.036) (Fig. 4). Putative therapeutic concentrations of ≥10,000 ng.hr/ml were only reached in 5 out of 8 subjects.

### Effect of BGC20-1531 on PGE_2_ related AEs

We found no difference in incidence of the AEs between the trial days (Table 2). No adverse events were reported during the pre-infusion period *T*_−75_–*T*_0_ except one participant who had an asymptomatic T-way inversion on ECG during pre-infusion period. The finding was defined by cardiologist as non-specific, but a decision was taken to exclude the participant from further experiments.

**Table 2:** Adverse events reported and recorded during the in-hospital period

	Placebo plus PGE_2_	BGC20-1531 200 mg plus PGE_2_	BGC20-1531 400 mg plus PGE_2_
Headache	6	6	7
Nausea	2	0	2
Photophobia	1	2	3
Phonophobia	0	0	0
Flushing	8	8	7
Heat sensation	6	5	5
Palpitation	4	7	4
Low back pain	1	0	1
Tightness in chest	4	3	2
Stiff muscles	2	1	2
Chills	1	1	0
Increased mucus production in throat	3	2	2
Parasthesia lips/fingers/arm	1	1	2
Local irritation around injection place	1	3	1
Low abdominal pain which mimics menstruation	1	1	1
Urge to void	3	1	2
Thirst	3	2	2
Face heat sensation	0	0	1

There was no difference between occurrence of AEs on placebo and BGC20-1531 200 mg pretreatment and BGC20-1531 400 mg pretreatment (*P* > 0.05, McNemar test)

## Discussion

To our knowledge, this is the first study where a potent and selective EP_4_ receptor antagonist, BGC20-1531, has been tested in a human model of headache. The main result was that the specific EP_4_ receptor antagonist did not prevent PGE_2_ induced headache in normal volunteers in this study.

PGE_2_ plays an important role in the regulation of cerebral haemodynamics [19]. In vitro studies have shown that PGE_2_ induces dilatation of human MCA and MMA [6, 12]. Similar results have also been obtained in cerebral and cranial arteries of animals [12, 20, 21]. However, in vivo animal studies have yielded conflicting results. An *open* cranial window model demonstrated that topical application of PGE_2_ caused dilatation of small and large pial arterioles in cat [22]. In a closed cranial window model, it caused dilation of the pial arteries in newborn pigs [23]. PGE_2_-induced dilatation of canine common carotid artery was also reported [12]. In the closed cranial window model, intracarotid administration of PGE_2_ caused dilatation of dural arteries but not pial arteries [20]. The conflicting data on whether or not PGE_2_ causes dilatation of arteries in different animal’s models may critically depend on the availability of PGE_2_ directly at the smooth muscle receptors, which is certainly different between luminal and abluminal administration.

### PGE_2_-induced headache and EP_4_ receptor antagonist

We have previously demonstrated in a double blind randomized crossover experiment, that intravenous PGE_2_ induces headache in healthy subjects [13]. Mean headache data recorded on the placebo day in the present study were in agreement with our previous study [13]. Given that the EP_4_ receptor antagonist, BGC20-1531 is highly selective [12], doses used in the present study should almost completely block EP_4_ receptors [12]. We therefore expected amelioration of PGE_2_-induced headache. However, two single doses of BGC20-1531 did not prevent the PGE_2_-induced headache. Oral dosing with BGC20-1531 (200 and 400 mg) in healthy volunteers has resulted in consistent plasma exposure in previous clinical studies with relatively low inter-subject variability (unpublished observations; single ascending dose study *C*_max_ 9,850 ± 2,900 and 22,700 ± 5,500 ng/ml at the 200 and 400 mg dose, respectively). The pharmacokinetic profile in the current study showed that exposure to BGC20-1531 was more variable (*C*_max_ 11,850 ± 5,800 and 21,100 ± 11,600 ng/ml at the 200 and 400 mg dose, respectively) and reached putative therapeutic concentrations in 5 out of 8 subjects (<10,000 ng hr/ml). However, results from the five subjects with sufficient plasma exposure did not indicate an effect of BGC20-1531.

It has previously been shown that other EP receptor subtypes such as EP_1_ [24, 25], EP_2_ [24, 25], EP_3_ [25], EP_3_A/α [24], EP_3_B [24], EP_3_β [24], EP_3_C [10] are expressed in sensory neurons and are involved in PGE_2_-induced sensitization [10, 24, 26, 27] and hyperalgesia [28]. Moreover, EP_1_ receptor antagonist GSK345931A attenuated hypersensitivity in a dose related manner in a preclinical model of inflammatory pain [29] and EP_3_ receptor knockout mice had reduced licking responses in the second phase of the formalin assay [30]. Aside from the possible activation of the other EP receptor subtypes in the presence of blocked EP_4_ receptors, PGE_2_ may also stimulate release of other vasoactive substances. It has been shown that EP_2_ receptor selective agonist, butaprost, as well as EP_3 _> EP_2_ receptor agonist, misoprostol, stimulate release of the vasodilator neuropeptide, calcitonin-gene related peptide (CGRP), which is well-known to be involved in the pathogenesis of neurovascular headaches [31]. Thus, we believe that involvement of the other PGE_2_ receptors subtypes is the most likely explanation of our headache results. Although due to the PK variability and low exposures noted with BGC20-1531, this does not preclude an involvement of the EP_4_ receptor subtype.

### EP_4_ receptor expression and EP_4_ receptor mediated dilatation of the intra- and extracerebral vessels

In our previous study on PGE_2_ induced headache in healthy subjects, we showed dilatation of MCA (13.9%, mean change from baseline) and STA (23.5%). The current vascular data, recorded on the placebo day were in agreement with this study [13]. It is well reported that PGE_2_-induced dilatation of cerebral blood vessels is mediated via EP_2_ and EP_4_ receptors [5, 32]. To our knowledge, very few immunohistochemical studies have reported the distribution of EP_4_ receptors in human vasculature. EP_4_ receptors, but not EP_2_ receptors are highly expressed in human pulmonary vein [33] and human renal artery [34] whereas EP_4_ and EP_2_ receptors show low expression in human pulmonary artery [33]. No data are available at present to show the distribution of EP_4_ and EP_2_ receptors in human intra- or extracranial arteries or in radial artery. Previous *in vitro* studies have reported that PGE_2_-induced dilatation of both human and animal isolated MCA [6, 12, 20] and MMA [12, 20] can be abolished by BGC20-1531 [6, 12, 20]. Furthermore, a specific EP_2_ receptor agonist causes no dilatation during stimulation [6, 20]. This suggests that PGE_2_-induced dilatation of those vessels is mediated through EP_4_ receptors.

In the present study, EP_4_ receptor antagonist BGC20-1531 did *not* prevent PGE_2_-induced velocity drop of MCA velocity and thereby dilatation of MCA. Interestingly, exploratory ANOVA analysis revealed statistical changes over time in V_MCA_ on the placebo day, but not on the active treatment day. Although there was no statistical effect of BGC20-1531on PGE_2_ responses, we found a modest trend of less velocity drop (Fig. 2). A weak antagonist effect could be due to low permeability of BGC20-1531 through the blood brain barrier and/or by the activation of EP_2_ receptors by PGE_2_. The prolonged and increased dilatation of STA after 200 mg BGC20-1531 is difficult to explain. It is possible that EP_2_ receptors are responsible for the dilating effect of PGE_2_ in the STA. The EP_2_ receptor has a shorter cytoplasmatic carboxyl terminus [35–37] and therefore, undergoes less internalization [38] and desensitization [39] after exposure to PGE_2_ compared to the EP_4_ receptor. In contrast to EP_4_, EP_2_ remains sensitive to metabolites of PGE_2_ [39, 40]_._ Hence, the activation of EP_2_ receptors could both prolong and intensify dilatation of STA. Future studies on EP_2_/EP_4_ receptors distribution in human STA, MCA and MMA may clarify these issues.

In conclusion, the selective blockade of EP_4_ receptors did not prevent PGE_2_ induced headache or vasodilatation. It should be noted that the present study was sufficiently powered to demonstrate effect based on previous study [41], however the low exposures of BGC20-1531 in 3 out of 8 volunteers may have contributed to the negative outcome in this study. Furthermore, we cannot exclude the PGE_2_-induced activation of the other EP receptors as well as possible low BBB permeability of the EP_4_ receptor antagonist. Therefore, further investigations of both PGE_2_ and EP_4_ receptors role in the pathogenesis of the neurovascular headache are hence worthy.
